# Nanomaterials for cancer therapy: current progress and perspectives

**DOI:** 10.1186/s13045-021-01096-0

**Published:** 2021-05-31

**Authors:** Zhe Cheng, Maoyu Li, Raja Dey, Yongheng Chen

**Affiliations:** 1 Department of Oncology, NHC Key Laboratory of Cancer Proteomics, Laboratory of Structural Biology, Xiangya Hospital, Central South University, Changsha, 410008 Hunan China; 2 National Clinical Research Center for Geriatric Disorders, Xiangya Hospital, Central South University, Changsha, 410008 Hunan China; 3 Department of Nucleotide Metabolism and Drug Discovery, The Hormel Institute, University of Minnesota, Austin, MN 55912 USA

**Keywords:** Nanomaterial, Cancer therapy, Tumor microenvironment, Exosome, Blood–brain barrier, Drug delivery, Protein corona

## Abstract

Cancer is a disease with complex pathological process. Current chemotherapy faces problems such as lack of specificity, cytotoxicity, induction of multi-drug resistance and stem-like cells growth. Nanomaterials are materials in the nanorange 1–100 nm which possess unique optical, magnetic, and electrical properties. Nanomaterials used in cancer therapy can be classified into several main categories. Targeting cancer cells, tumor microenvironment, and immune system, these nanomaterials have been modified for a wide range of cancer therapies to overcome toxicity and lack of specificity, enhance drug capacity as well as bioavailability. Although the number of studies has been increasing, the number of approved nano-drugs has not increased much over the years. To better improve clinical translation, further research is needed for targeted drug delivery by nano-carriers to reduce toxicity, enhance permeability and retention effects, and minimize the shielding effect of protein corona. This review summarizes novel nanomaterials fabricated in research and clinical use, discusses current limitations and obstacles that hinder the translation from research to clinical use, and provides suggestions for more efficient adoption of nanomaterials in cancer therapy.

## Background

Despite significant advances in medical science and technology, cancer remains a disease with limited treatment approaches. Metastasis and recurrence of cancer contribute a lot to disability and mortality, and the exact mechanisms remain to be illuminated [1, 2]. Cancer is generally considered as the consequence of gene mutations [3]. In 2018, there were an estimated 18.1 million new cancer cases and 9.6 million deaths were caused by cancer [4]. According to the Global Cancer Observatory (GCO), approximately 30 million cancer patients will die from cancer each year by 2030 [5]. In addition to the high mortality of cancer, the economic burden on families of cancer patients and society is enormous. Therefore, efforts on cancer prevention, diagnosis and treatment are of great importance.

Cancer is characterized by abnormalities in mechanisms that regulate cell cycle, leading to the survival and proliferation of malignant cancer cells. Signaling pathways are usually altered when cancer occurs. Inhibition of physiological apoptosis contributes to cancer development as well as resistance to radiotherapy and chemotherapy [6]. Inflammation and immune system disorder are also related to cancer. Traditional tumor staging (AJCC/UICC-TNM classification) is based on tumor burden (T), presence of cancer cells in draining and regional lymph nodes (N), and tumor metastases (M). Cancers can also be classified according to organs of origin, such as lung, colon, breast, head and neck, kidney, bladder, prostate, ovary, or various cancer cell types [7].

Current cancer diagnosis approaches include imaging methods, laboratory tests, and morphological analysis of tissues and cells, which is usually considered highly reliable in most cancer diagnosis [8]. Pathological characteristics such as immunohistochemical (IHC) analysis, histological alterations, mutational and molecular genetics analysis also help cancer diagnosis [9]. Common cancer treatment consists of surgical resection, chemotherapy, radiotherapy, and biological therapy. Surgery is an effective measure to remove malignant solid tumors, especially in an early stage of cancer development. Combined therapy involves several therapies such as surgery, chemotherapy, and radiotherapy. The application of chemotherapy has been popular over the years for its simplicity and convenience in treating cancer patients [10, 11].

Chemotherapy is effective for various cancers, including acute myelogenous leukemia, acute lymphoblastic, Hodgkin’s and non–Hodgkin’s lymphoma, small cell lung cancer, germ cell cancer, ovarian cancer and choriocarcinoma [12]. However, the indiscriminate cytotoxicity of chemotherapy causes undesirable side effects, as chemotherapy can also inhibit rapid-growing tissues and cells including hair follicles, gastrointestinal tract cells, and bone marrow. The use of chemotherapy also induces multi-drug resistance (MDR) and has potential association with cancer stem cells (CSCs). Cytotoxic chemical drugs used in chemotherapies are non-specific and heterogeneous in terms of distribution that contribute to MDR in the treatment process [13, 14]. This non-specificity impedes chemotherapy efficacy and impairs inhibition of tumor growth, metastasis and recurrence [15].

Current chemotherapy faces problems such as lack of specificity, cytotoxicity, short half-life, poor solubility, occurrence of multi-drug resistance and stem-like cells growth. To overcome these disadvantages, nanomaterial-based chemotherapy, targeted therapy, molecular therapy, photodynamic therapy (PDT), photothermal therapy (PTT), chemodynamic therapy (CDT), and sonodynamic therapy (SDT) are being used in cancer treatment. In addition, a substantial number of studies on variety of therapies such as molecular therapy, apoptosis regulations, immunotherapy, signal modification therapy, nucleic-acid-based therapy, and anti-angiogenesis therapy for the treatment of cancer have been done in recent years [16–18]. With the advent of nanotechnology nanomedicines used in cancer therapy can possibly reduce drawbacks of chemotherapy and an extensive research studies have been going on along this direction.

## Nanotechnology applied in cancer therapy

### Properties of nanomaterials

Medical nanotechnology uses materials with nanorange size, which is generally 1–100 nm. These materials are applied in the therapeutic drugs and devices design, manufacture [19]. As the size shrinks to nanoscale, many unique optical, magnetic and electrical properties emerge, making nanomaterials differ from traditional macromolecules. Typical nanomaterials possess several common characteristics: high surface-to-volume ratio, enhanced electrical conductivity, superparamagnetic behavior, spectral shift of optical absorption, and unique fluorescence properties. In the medical field, nanomaterials can be applied in drug transportation, controlled release. Increased permeability enabling crossing through biological barriers and improved biocompatibility are also noticeable features [20].

These particular properties of nanomaterial suggest it can be utilized in cancer therapeutics. The high surface-to-volume ratio of some nanomaterials can assemble with biomolecules or residues, which can enhance the specificity of chemical drug complex in targeted therapy, thereby enhancing the efficacy of nanomaterial-based treatment while reducing its toxicity to normal cells [21]. PDT and PTT are two treatment methods related to optical interference. In PDT, a photosensitizer is accumulated in cancerous sites; when irradiated with certain wavelength light, singlet oxygen and other cytotoxic reactive oxygen materials are generated, causing apoptosis and/or necrosis [22]. PTT uses materials that possess high photothermal conversion efficiency to elevate the temperature of targeted cancerous areas, leading to cancer cell death. PDT and PTT are emerging cancer treatment methods with great potential, and materials used in these two therapies are under intensive research. Some nanomaterials can be used in PDT and PTT because of their unique fluorescence properties [23]. The superparamagnetic behavior of nanomaterials provides several usages for cancer diagnosis and treatment. A common nanomaterial, superparamagnetic iron oxide nanoparticles (SPION), has potential in cancer hyperthermia treatment due to its smaller size, higher targeting specificity, controllable releasing speed, and immune evasion capability [24].

### Progress of nanotechnology in targeted delivery

Targeted delivery is one of a major advantage of nanomaterial-based cancer therapy over free drugs. Recent progress has been made in targeted delivery based on nanomaterials. The idea of targeted delivery aims for precise targeting of specific cancer cells, and it is achieved by either passive targeting or active targeting. Enhanced permeability and retention (EPR) effect is used in passive targeting while active targeting is achieved by conjugating with antibodies, peptides, aptamers and small molecules. Compared with free drugs, targeted delivery helps reduce toxicity in normal cells, protect drugs from degradation, increase half-life, loading capacity, solubility [19, 25].

Through delicate design and modification, nano-drugs can maintain better specificity, bioavailability, less cytotoxicity to normal tissue, larger loading capacity, longer half-life period, and unique drug release patterns, overcoming disadvantages of conventional chemical therapy. During the past two decades, tremendous development in cancer pathology and nanoscience, technology, and industry (NSTI) created plenty of nanomaterials for cancer treatment and diagnosis.

**Fig. 1:**
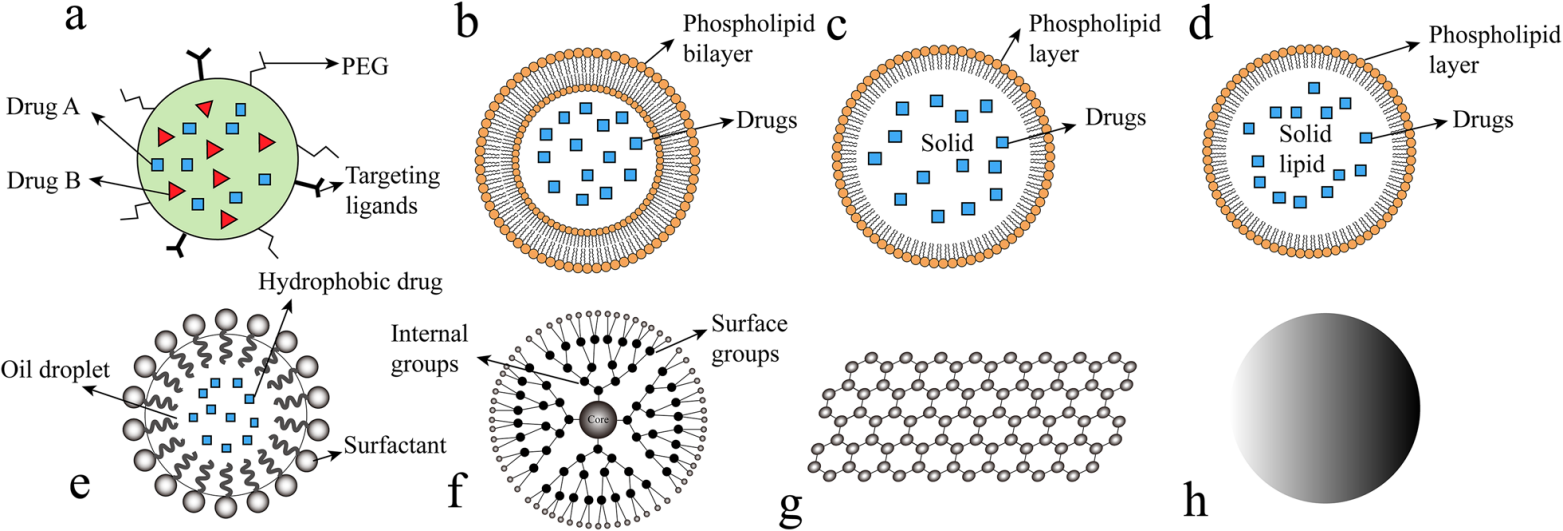
Categories of nanomaterials applied in cancer treatment. **a** Nanoparticles. **b** Liposomes. **c** Solid lipid nanoparticles. **d** Nanostructured lipid carriers. **e** Nanoemulsions. **f** Dendrimers. **g** Graphene. h Metallic nanoparticles. PEG, poly(ethylene glycol)

However, only a relatively small number of nano-drugs have been well developed and involved in clinical use. These nanomaterials can be generally classified into several categories (Fig. 1).

## Nanomaterials used for cancer treatment

### Nanoparticles

#### Polymeric nanoparticles

Nanoparticles are particles with size of nanoscale. Polymeric nanoparticles (PNPs), mAb nanoparticles, extracellular vesicles (EVs), metallic nanoparticles are broadly researched nanoparticles (NPs) (Table 1). PNPs are defined as colloidal macromolecules with submicron size of 10–1000 nm. As drug carriers, PNPs carry chemical drugs and achieve the sustained release to targeted cancerous sites [26]. Drugs are encapsulated or attached to the surface of nanoparticles thus forming a nanocapsule or a nanosphere. The ingredients of nanoparticles have changed over the years. Initially, nonbiodegradable polymers such as polymethyl methacrylate (PMMA), polyacrylamide, polystyrene, and polyacrylates were used to fabricate nanoparticles [27, 28]. To avoid toxicity and chronic inflammation, polymeric nanoparticles made by these materials shall be cleared up in time. The accumulation of these types of polymer-based nanoparticles in tissues to a toxic level caused due to the difficulty to get them degraded, excreted, or physically removed have now been solved. Biodegradable polymers have been manufactured to reduce toxicity, improve drug release kinetic patterns and increase biocompatibility. These polymers include polylactic acid (PLA), poly(lactic-co-glycolic acid) (PLGA), poly(amino acids) [29], poly(ε-caprolactone) (PCL), and natural polymers consist chitosan, alginate, gelatin and albumin. These improved polymeric nanoparticles have special advantages due to their properties and structures. For volatile pharmaceutical agents, PNPs help increase stability. For chemical drugs, PNPs provide optional administration methods such as oral and intravenous and higher loading ability compared to free drugs. The ability that protects drugs from degradation helps minimize undesired toxicity to normal tissues; for instance, PNPs loaded with cisplatin such as dexamethasone or α-tocopheryl succinate have been employed in chemotherapy, which prevents cisplatin-induced ototoxicity [30].

**Table 1:** Summary of NPs in development or research stage for cancer therapy

Modification	Payload	Therapies involved	Target cancer model	Outcome	References
PLGA NP	PTX	Chemotherapy	Human prostate cancer lines PC3	Drug delivery efficiency was highly improved compared with free PTX	[31]
PEG, transferrin modified NP	Nucleic acids	Nucleic-acid-based therapy	Human prostate cancer lines PC3 Chronic myelogenous leukemia cells K562	Showed higher efficiency over untargeted particles when transfect K562 leukemia cells	[32]
Tmab modified NP	Docetaxel	Targeted therapy, chemotherapy	Human HER2-postive BT474 cells and HER2-negative MCF7 cells	Increased cytotoxicity in HER2-positive BT474 cells but not in HER2-negative MCF7 cells	[33]
Tmab modified NP	Paclitaxel	Targeted therapy, chemotherapy	Human HER2-postive breast cancer cell lines: BT474, SK-BR-3; HER2-negative cell line: MDA-MB-231	Better treatment efficacy and lower cytotoxicity to human breast epithelial cell control were exhibited	[34]
PLGA NP	Alantolactone Erlotinib	Targeted therapy	Human pancreatic cancer cell lines PANC-1 and Patu8988T	The synthesized NP induced significant cancer cell apoptosis and showed anticancer effect	[35]
Exosome	Doxorubicin	Chemotherapy	Human breast cancer cells MDA-MB-231; Mouse ovarian cancer cells; Breast and ovarian cancer mouse models	Cytotoxicity of doxorubicin was enhanced and drug accumulation in mouse heart was avoided	[36]
AuNP encapsulated IONPs/Ag cores	IONPs/Ag	PTT	C57BL/6 implanted with B16-F10 melanoma tumors	The gold NP complex acted well as MRI T2 contrast agent and was an effective PTT agent	[37]
Trithiol-terminated poly-methacrylic acid modified nanorods	Fe_2_P	SDT, PTT	Human cervix cancer cells HeLa; non-cancerous mouse fibroblast cells L929	The nanorod was biocompatible and showed ultrasound, photothermal synergistic therapeutic properties	[38]

GSH, Glutathione; NP, Nanoparticle; PLGA, poly (lactic-co-glycolic acid); PTT, Photothermal therapy; SDT, Sonodynamic therapy; Tmab, Trastuzumab

There are two main drug delivery methods: passive targeting and active targeting (Fig. 4a). A dense extracellular matrix causes difficulty for drugs to infiltrate while over-activated angiogenesis poses a certain advantage objectively known as EPR. When tumor grows, plenty of nutrition and oxygen are needed; in the meantime, tumor-induced angiogenesis generates many immature vasculatures that suppresses lymphatic drainage [39]. These leaky blood vessels make it possible for chemical drugs to penetrate into cancerous sites. However, the size of drugs is crucial as regular particles are not small enough to percolate through cancerous cells. On the contrary, nanoparticles and related chemical drug vehicles can easily penetrate targeted sites and accumulate because of attenuated lymphatic drainages [40].

PNPs share the common property of high surface-to-volume ratio as nanoscale particles, making it convenient to attach targeting polymers onto the surface. A proven research has shown that bioavailability can be enhanced by coating polymers with polysorbates, utilizing polysorbates surfactant effect through endothelial cell membrane solubilization and fluidization. Surface coating helps PNPs interact with blood–brain barrier(BBB) endothelial cell membranes and facilitate endocytosis [41, 42]. As novel nanocarriers function differently from conventional chemical therapy, polymeric nanoparticles can deliver several sorts of chemicals to target sites including anti-cancer drugs, small interfering RNAs (siRNA), radionuclide, and specially designed polymeric nanoparticles possessing the ability to react to ultra-sound. Fluorescent polymeric nanoparticles are used as theragnostic tools. Theragnostic is a strategy combining diagnosis and treatment at the same time. Fluorescent polymeric nanoparticles (FNPs) have been identified as novel theragnostic materials in recent years. To achieve both diagnostic and therapeutic functions, nanomaterials with complex structures are fabricated. A FNP usually consists of fluorescent proteins, biocompatible biopolymers, inorganic quantum dots, and organic dyes [43]. In addition to tumorous imaging, drugs can be loaded by *π*–*π* bond or hydrophobic interactions in fluorescence assays that eventually enhances the anti-cancer efficacy of nanomedicine [44]. In siRNA delivery, cyclodextrin polymer (CDP)-based nanoparticles improve delivery efficacy in vivo [45]. Research studies have shown that transferrin modified adamantane-Polyethylene glycol (AD-PEG) and adamantane-PEG-transferrin (AD-PEG-Tf) are appropriate to deliver nucleic acid in vivo [32, 46]. Nanoparticles can be used to encapsule radionuclide such as I125 via electrophilic aromatic substitution which is in high radiochemical yields. Through this straightforward way, radionuclide can be stored in the stable core [47, 48]. Dey [49] developed a self-assembling peptide/protein nanoparticle with the size only 11 nm in diameter and it exhibited good biocompatibility and stability in vivo, indicating it should be suitable for drug delivery in cancer treatment. Ultrasound sensitive polymeric nanoparticles have emerged as an efficient tool for cancer diagnosis and treatment. Several uses of ultrasound interactive nanoparticles have been implemented. Use of ultrasound in NP manufacture helps enhance efficacy of drug delivery, therefore leads to reduction of side effects through improved traversing ability to overcome the barriers in cancer therapy. These barriers include endothelial blood vessels [50], tissue endothelium, interstitium, nuclear membrane and BBB [51, 52]. Since ultrasound can result in a thermal effect that may eventually break the nanoparticles, ultrasound can also be used as a preset trigger through which chemical drugs can be released under control [53]. However, the polymeric nanoparticle has its disadvantages: evidence shows that some polymeric nanoparticles undergo toxic degradation and toxic monomers aggregation thereby needing further studies for their improvement in fabrication and chemical properties [54].

#### mAb nanoparticles

Recent progress has been made in mAb nanoparticles. In targeted therapies, monoclonal antibodies (mAbs) are vastly used for their specific targeting ability and anti-tumor effect. Moreover, in recent years, mAbs are used in designing novel anti-tumor nanoplatforms and has been forefront in the field.

To further increase therapeutic efficacy of anticancer drugs, mAbs are conjugated with cytotoxic drugs, this is termed as antibody–drug conjugates (ADCs); With specific antigens expressed differently in cancerous cells and normal cells guiding the drug complex, better specificity and less toxicity can be achieved [55]. Trastuzumab (Herceptin) is a mAb used to treat breast cancer with positive expression of human epidermal growth factor receptor 2 (HER2). Research using trastuzumab (Tmab) in ADC system have been conducted, and the result shows improved therapeutic efficacy compared with Tmab alone [56]. Abedin et al. fabricated an antibody–drug nanoparticle, which consists of a core loaded with paclitaxel (PTX) and a surface modified with trastuzumab. Two HER2-positive cell lines and one HER2-negative cell line were treated with this novel NP, PTX, and trastuzumab separately and the result was inspiring: NP complex showed better anti-tumor efficacy than PTX or trastuzumab alone, and relatively lower cytotoxicity in human breast epithelial cell control was observed in NP complex group [34] (Fig. 4a). Trastuzumab NPs based on ADC mechanism are promising nanoplatforms in cancer therapy and vast research are being conducted [57–59].

#### Extracellular vesicles

EVs are bilayer phospholipid vesicles with the size typically range from 50 to 1000 nm [60]. EVs are secreted continuously by various cell types and differ in size, origin, and content. Based on the origin, EVs are classified into three major groups: exosomes, microvesicles and apoptotic bodies [61, 62]. Exosomes are 40–200 nm nano-scale particles. EVs contain protein, RNA and DNA and are involved in long-distance communications [63]. Because exosome membrane contains similar lipids and molecules to their origin cells, exosome NPs can escape the immune surveillance and internalize smoothly with target cells, and exosome NPs are natural carriers to be combined with existing anti-tumor compositions and methods.

Gene therapy utilizes DNAs/RNAs in cancer treatment to take effect. Several approaches are explored in gene therapy, including restoring mutated proto-oncogene such as p53 [64], inhibitor of growth 4 (ING4), phosphatase and tensin homolog (PTEN) [65] and gene editing using clustered regularly interspaced short palindromic repeats (CRISPR)-associated proteins (Cas) system that disables key oncogenes [66, 67]. RNA interference (RNAi) can be caused by small RNAs such as siRNAs and microRNAs (miRNAs). RNAi contributes to physiological and pathological process. Research that targeting oncogenic mRNAs by siRNA is under evaluation [68]. Gene therapy can also induce cell death by delivering transgene or cell death-triggering gene to tumor cells [69].

Researchers have utilized exosomes as nanoparticle platforms to delivery nucleic acids, small molecules, and proteins [36, 70] (Table 2). Hadla et al. used exosomes loaded with DOX (exoDOX) to treat human breast cancer cells and the result showed that compared with free DOX, exoDOX enhances the cytotoxicity of doxorubicin and avoid drug accumulation in the heart [36]. Exosomes can be engineered for targeted delivery in cancer treatment. A macrophage-derived exosome was modified with aminoethylanisamide-polyethylene glycol (AA-PEG) moiety, and the AA-PEG exosome was loaded with PTX. The engineered exosome showed improved therapeutic outcomes in pulmonary metastases mouse model [71]. Jeong et al. utilized exosomes to deliver miRNA-497 (miR-497) into A549 cells, and the result showed that tumor growth as well as expression of associated genes were suppressed, indicating this exosome-mediated miRNA therapeutic can be used in targeted cancer therapy [72]. Compared with synthetic NPs, exosome NPs possess inherent biocompatibility, higher chemical stability and the ability to manage intercellular communications. However, there are obstacles of exosome NP application, such as lack of uniform criteria of exosomal isolation and purification, unclear mechanism of exosome in cancer treatment, heterogeneity and difficulty preserving [73–75].

**Table 2:** EVs used as nanocarriers in cancer therapy

Nanocarrier	Pharmaceutical ingredients	Disease	Outcome	References
AA-PEG modified exosome	Paclitaxel	Lung cancer	Exhibited high loading capacity, better accumulation in cancer cells and improved therapeutic outcomes	[71]
Exosome	Doxorubicin	Osteosarcoma	Anti-tumor effect was enhanced while cytotoxicity to myocardial cells was reduced compared to free DOX	[76]
Exosome	miR-497	Lung cancer	Tumor growth as well as expression of associated genes were suppressed	[72]
Microvesicle	Therapeutic mRNA/protein	Schwannoma	The suicide therapeutic mRNA/protein loaded microvesicle converted the prodrug to active form and led to cancer cell death	[77]
EV	miR-101	Osteosarcoma	Osteosarcoma cell invasion and migration were suppressed after taking in miR-101 loaded EVs	[78]
Exosome-Liposome Hybrid NP	CRISPR/Cas9 system	/	The hybrid NPs can deliver CRISPR-Cas9 system in MSCs and might be used in cancer therapy	[79]
Exosome	Interferon-γ fusion protein	Prostate cancer	The exosomal vaccine induced immune response against prostate cancer derived exosomes and inhibited tumor growth	[80]

Cas, CRISPR-associated proteins; CRISPR, Clustered regularly interspaced short palindromic repeats; EV, Extracellular vesicle; MSC, Mesenchymal stem cell

### Lipid-based nanomaterials

Research on lipid-based nanomaterials is blooming and three main categories have been receiving great attention in current research and clinical trials: liposomes, solid lipid nanoparticles (SLNs), and nanostructured lipid carriers (NLCs). Liposomes were approved in 1965 and considered the first enclosed microscopic phospholipid bilayer nanosystem [81]. Liposomes are spherical vesicles composed mainly of uni-lamellar or multi-lamellar phospholipids, and the size of a liposome usually ranges from 20 nm to more than 1 um [82, 83]. A liposome generally has a hydrophilic core and a hydrophobic phospholipid bilayer. This kind of structure enables entrapment of both hydrophilic and hydrophobic drugs [84] depending on the pharmacokinetic properties of the drug. Liposomes with the typical structure encapsulate hydrophilic drugs within their aqueous core and hydrophobic drugs in the lipid bilayer. Drugs encapsulated within the central cavity of liposome are protected from environmental degradation during the circulation through human bloodstream [85]. The size and number of bilayers are two important parameters that affect the loading amount and half-life of drugs; therefore, liposomes can be classified into two types according to these two conditions: unilamellar vesicles and multilamellar vesicles (MLV). Unilamellar vesicles are further divided into small unilamellar vesicles (SUV) and large unilamellar vesicles (LUV). An onion-like structure is formed in multilamellar liposomes, while several unilamellar vesicles can be formed inside other vesicles and form multilamellar concentric phospholipid spheres separated by water molecules [86].

As per extensive research on nanocarriers, recent liposomes bear plenty of unique properties and characteristics; correspondingly, novel applications have emerged based on liposome materials. Three major issues have been discovered and dealt with over the development of liposomes. Breaking through biological barriers and avoidance of rapid clearance are problems researchers have been encountering. As referred to above, biological barriers have always been major technical obstacles for nanocarriers to overcome. Regarding liposomes, cells of the mononuclear phagocyte system (MPS) predominantly in the liver and spleen are acting as human guards and phagocytizing nanoliposomes. Modifying membrane is one of the major techniques to prolong liposome half-lives. Covering the membrane with proteins, peptides, polymers, or other molecules significantly enhances the ability to escape from the MPS system and therefore helps achieving longer liposomal half-lives [87]. This kind of liposomes was named “Stealth” liposomes. Later polyethylene glycol conjugated liposome was found to have a longer half-life compared to other modified liposomes. Based on this observation, PEG-liposomes loaded with doxorubicin (DOX) were used to treat Kaposi's sarcoma in HIV patients [88].

Drug-loading and controlled release of liposomes are also important issues that need attention in liposome nanocarrier design. For cancer chemotherapy, bioavailability affects drug efficacy. Compared to free DOX, DOX liposome has a lower bioavailability, indicating that improving the bioavailability should be considered when design liposomes [89]. Co-delivery and controlled release are two major applications of liposomes. Combinations with chemical drugs, metals, gene agents, and other chemotherapeutic agents have been formed. Overactivation of certain signaling pathways is one of the patterns of cancer occurrence, and drugs targeting these signaling pathways are applied. To achieve higher efficacy, researchers loaded a novel PEGylated liposomal with ncl-240 and cobimetinib which are small-molecule inhibitors of the phosphoinositide 3-kinase/mammalian target of rapamycin (PI3K/mTOR) pathway and mitogen-activated protein kinase kinase/ extracellular signal regulated protein kinase (MEK/ERK) pathway, respectively. The result showed that the cytotoxic effect was enhanced due to synergistic effects [90]. A novel liposome-encapsulated nanocarrier loaded with both irinotecan and floxuridine showed better efficacy in advanced solid tumors [91]. The delicate structure of a novel liposome with multiple layers enabled it to effectively load up to 3500 siRNA molecules in a single bilayer and codelivery of DOX, which demonstrated better DOX efficacy and shrink of tumor mass in breast cancer treatment [92]. Triggered release and target methods are extensively studied. As cancerous areas have an average 6.8–7.0 extracellular pH value, which is slightly more acidic than healthy tissue [93], liposomes can be designed to release drugs when reaching acidic cancerous areas. With a pH-sensitive material, carboxymethyl chitosan (CMCS) coated to the surface, the cationic liposome (CL) preloaded with sorafenib (Sf) and siRNA (Si) obtained pH-sensitive property. Results showed that sorafenib release was enhanced and cellular uptake was increased at the pH of 6.5 [94]. Other than pH-responsive property, liposomes can also be fabricated with enzyme-responsive, redox-responsive, light-responsive characteristics, depending on tumor microenvironment (TME) and drug properties [95]. TME is the concept of the environment in which tumor cells are living. TME facilitates tumor growth, invasion, migration, angiogenesis, inflammation and it is related with drug resistance [96, 97]. The common characteristics of TME include the presence of EPR, hypoxia (lack of oxygen), acidosis (low pH), extensive angiogenesis, and tumor-associated immune cells that help the immune escape of cancer cells [98]. In general, liposomes' advantages are protecting loaded drugs from enzyme degradation, low toxicity, biocompatibility, flexibility, superior biodegradability, and non-immunogenicity [99]. However, application of liposome is limited due to disadvantages such as short shelf life, low encapsulation efficacy, dissatisfying stability, rapid removal by MPS, cell adsorption, and intermembrane transfer.

SLNs are colloidal nanocarriers with a nanoscale of 1–100 nm. Because of the strict limits on the size, SLNs are referred to as the “zero-dimensional” nanomaterials, as they differ from other larger nanomaterials by at least one dimension in nanoscale. Unlike liposomes, the ingredients of SLNs include solid materials such as solid lipid, emulsifier, and water. Partial glycerides, triglycerides, fatty acids, waxes, steroids and PEGylated lipids are lipid used in SLNs [19]. In terms of structure and function, there are similarities and differences between SLNs and conventional liposomes. The similarities are the lipidic outer layer and delivery function of chemical drugs. Unlike traditional liposomes which consist of lipid bilayers that surround an aqueous pocket, some SLNs do not have a contiguous bilayer; instead a micelle-like structure is formed and drugs are encapsulated in a non-aqueous core [100]. Lipid components of SLNs are solid at body temperature, and SLNs have better stability and prolonged release than liposomes. However, SLNs have limitations that are unpredictable gelation tendency and inherent low incorporation rates because of their crystalline structure [101].

NLC carrier was developed in the past two decades as an improved generation of both liposome and SLN. To improve stability and loading capacity while maintaining intrinsic protection function, biocompatibility, and non-immunogenicity, NLCs are designed as a system consisting of a core matrix loaded with both solid and liquid lipids. NLCs can be administrated through multiple methods: oral, parenteral, inhalational, and ocular. As many drug compounds used in cancer treatment are lipophilic, NLCs have gained lots of attention in recent years [102].

### Nanoemulsions

Nanoemulsions (NE) are colloidal nanoparticles made of aqueous phase, emulsifying agents as well as oil [103]. The size of nanoemulsion ranges from 10 to 1000 nm. Nanoemulsions are widely used drug nanocarriers, usually solid spheres with amorphous and lipophilic surface that exhibit negative charge. As nanoemulsions are heterogeneous mixtures containing oil droplets in aqueous media, nanodroplets are distributed with small size, and three typical types of nanoemulsions can be formulated: (a) water in oil nanoemulsion system in which water is dispersed in an aqueous medium; (b) oil in water nanoemulsion system in which oil is dispersed in an aqueous medium; (c) bi-continuous nanoemulsion [103]. Nanoemulsions have several advantages over most lipid-based nanomaterials and nanoparticles: optical clarity, thermodynamic stability, large surface area, convenience in manufacture, biodegradability, and ideal drug release profile [104]. Membrane modified nanoemulsions have been extensively studied. Co-delivery by nanoemulsions is one of the methods to enhance bioavailability and drug efficacy. The test results of a NE drug carrier system loaded with spirulina polysaccharides and PTX showed that it could improve the anti-tumor effect of PTX by regulating immunity through Toll-like receptor 4/nuclear factor kappa B (TLR4/NF-κB) signaling pathways [105]. A nanoemulsion system consisting of temozolomide, rapamycin, and bevacizumab was established to treat advanced melanoma. Through parenteral administration, enhanced cytotoxicity against melanoma cells and improved inhibition of tumor relapse, migration and angiogenesis were observed in vitro human and mouse cell models [106](Fig. 4b).

Nanoemulsions can also be applied to immune therapy by loading certain immune-stimulation moiety. Cytokine Interferon gamma (IFN-γ) was loaded in a modified nanoemulsion to stay stable in extreme temperature changes for three months. The test results showed that this NE suppressed cell viability of MCF-7 human breast cancer cells and induced cellular activity of phagocytes, suggesting a promising potential in cancer treatment [107] (Fig. 4c). One application that gains plenty of attention is using NE as a strategy to overcome MDR. In MDR cancer cells, ATP-binding cassette transporters (ABCs) are responsible for part of MDR occurrence. MDR transporters expressed by ABCs cause resistance to anticancer drugs. P-glycoprotein (P-gp) is the first identified ABC transporter encoded by ABC1 gene which possesses function of pumping colchicine, vinblastine, etoposide and paclitaxel (PCX) from the cell [13]. To overcome this obstacle, a novel NE co-delivering baicalein and paclitaxel was fabricated by Meng and colleagues. By co-encapsulating these two drugs, oxidative stress was elevated, thereby providing a suitable strategy to improve cell sensitivity to paclitaxel. Results showed that reactive oxygen species (ROS) was increased, cellular glutathione (GSH) was decreased, caspase-3 activity was enhanced in MCF-7/Tax cells, and an *in*-*vivo* study showed that baicalein-paclitaxel NE exhibited a superior antitumor efficacy than conventional paclitaxel formulations [108, 109]. These studies clearly exhibit the potential benefit of using specially manufactured NEs in MDR management.

Despite potential benefit NEs possess, there are challenges to clinical application. The production of NEs usually involves high temperature and pressure. Therefore, not all starting materials are suitable in NE application. This is one of the obstacles in applying NEs to massive commercial production. In NE preparation, high-energy methods such as homogenizer and microfluidizer are used, which makes NE costlier than other conventional formulation. Because of lack of understanding of chemistry in NE production, detailed research should be conducted about component interaction and NE metabolism in human body to assess the safety in clinical use [104].

### Dendrimers

Dendrimers are a kind of unique macromolecules with hyperbranched defined architecture. The most apparent characteristic of dendrimers is their highly branched and easily modifiable surfaces. The size of these dendrimer polymers is ranging mainly from 1 to 10 nm, while some specially fabricated large dendrimers can reach up to diameters of 14–15 nm [110, 111]. Three major structural parts form the dendrimer molecules: central core that loads theragnostic agents through noncovalent encapsulation, branches that form the interior dendritic structure, and the exterior surface conjugated with functional surface groups. Several dendrimers have been developed for cancer therapeutics: polyamidoamine (PAMAM), PPI (polypropylenimine), PEG (poly(ethylene glycol)), Bis-MPA (2,2-bis(hydroxymethyl) propionic acid), 5-ALA (5-aminolevulinic acid), and TEA (triethanolamine) [112].

Due to specific structure, dendrimers have unique features over other nanomaterials: defined molecular weight, versatile adjustable branches, narrow polydispersity index, superior solubility and bioavailability of hydrophobic drugs. Cationic dendrimers with positively charged surfaces can form complexes with nucleic acids; therefore, dendrimers can be used as efficient nucleic acid nanocarriers. PAMAM and PPI are two widely studied dendrimers with various application strategies. A PAMAM dendrimer/carbon dot nanohybrid was designed to achieve MDR management and cancer cell monitor simultaneously via fluorescence imaging. Two complexes were manufactured separately. The first part was a CDs/DOX complex consisting of blue-emitting carbon dots (CDs) and anticancer drug DOX through non-covalent interactions. The other part was G5-RGD-TPGS, which consists of generation 5 (G5) PAMAM dendrimers, targeting ligand cyclic arginine-glycine-aspartic (RGD) peptide and drug efflux inhibitor d-α-tocopheryl polyethylene glycol 1000 succinate (TPGS). Two parts were connected by electrostatic interaction and formed a dual drug-loaded nanohybrid system. In vitro fluorescence was achieved by the luminescence of CDs, and targeting specificity was achieved by the presence of RGD ligands that targets αvβ3 integrin receptors overexpressed in cancer cells [113]. The results showed that TPGS had a significant inhibitory effect on cancer cells. The Co-delivery ability of dendrimer can also be used in delivering completely different materials. DOX is commonly used to treat colon cancers. The tumor necrosis factor-related apoptosis-inducing ligand (TRAIL) is a crucial factor in the apoptotic pathway, capable of binding to death receptors 4 and 5 (DR4 and DR5), which are overexpressed in various cancer cells. Pishavar group encapsulated DOX and TRAIL plasmid in a dendrimer nanocarrier, which exhibited a stronger antitumor effect than modified carriers containing DOX or TRAIL plasmid alone [114]. A PAMAN nanocarrier based on dendrimer was synthesized and used for chemotherapy combined with photothermal treatment of liver cancer cells. Though PAMAN dendrimers without modification have disadvantages such as low transfection efficiency, inefficient cellular internalization and instability of encapsulation [115], the nanomaterial has competitive contrast properties, showing great potential in combination therapy [116].

### Carbon nanomaterials

Carbon nanomaterials (CNMs) are a kind of nanosized material with many categories based on carbon element. CNMs have been widely used in many industrial and medical fields because of their unique electronic, thermal, optical, and mechanical properties (Table 3). In cancer theragnostic applications, CNMs are considered more biocompatible and safer than metal-based nanomaterials [117, 118]. CNMs can load chemical drugs through *π*–*π* stacking or hydrophobic interactions due to inherent hydrophobic nature, making CNMs as efficient drug delivery platforms [119, 120]. Several carbon nanomaterials have been massively studied in cancer treatment: graphenes, fullerenes, carbon nanotubes (CNTs), carbon nanohorns (CNHs), carbon quantum dots (CQDs) and graphyne (GDY). Although all these materials are based on carbon elements, the morphological structure, properties, and functions of these nanomaterials vary greatly.

**Table 3:** Recent studies on CNMs for cancer therapy

Type of materials	Loaded drug	Feature	Anti-cancer effect	References
rGO	PTX	Phosphorylcholine oligomer grafted perylene-modified	Cytotoxicity of PTX against SGC7901 tumor cell line was improved compared with free PTX	[166]
rGO	DOX	Folic acid-conjugated	Enhanced specificity and cytotoxicity of DOX to MBA-MB 231 human breast cancer cells	[167]
rGO	MTX	Gold NPs-coated	Activity of MTX on MCF-7 was improved compared to free MTX	[168]
GO	MTX	Dopamine-conjugated	Capacity of MTX targeting dopamine receptors expressing cancer cells was enhanced	[169]
GO	DOX	Carboxymethyl cellulose-functionalized	DOX was released pH-dependently and showed good antitumor activity and biocompatibility without no obvious cytotoxicity	[170]
Fullerene	C_60_ (OH)_22_	Targeting at cancer stem cells	Biological communication of stem cells and tumor cells was inhibited	[171]
Fullerene	Gd@C_82_ (OH)_22_	Angiogenesis	10 proangiogenic factors were downregulated in mice model	[172]
CNT	Hydrazine–SWNT–DOX	pH-sensitive drug release	Great cytotoxicity toward HepG2 tumor cells with high weight loading	[173]
CNT	Chitosan–MWCNT–DOX	Used in photothermal/chemotherapy	Sustained release of DOX and significant hyperthermia exhibiting remarkably enhanced anti-tumor efficacy	[174]
CQD	CQD–mesoporous silica nanoparticle–DOX	pH-sensitive drug release	80% DOX load released at pH 5.0 and a remarkably enhanced anti-tumor efficiency was exhibited	[175]

CNTs, Carbon nanotubes; CQDs, Carbon quantum dots; DOX, Doxorubicin; GO, Graphene oxide; MTX, Methotrexate; MWCNTs, Multiwalled carbon nanotubes; PTX, Paclitaxel; rGO, Reduced graphene oxide

Graphene is a two-dimensional crystal with sp2 -hybridized carbon sheet which possesses remarkable mechanical and electronic properties. It has also been heavily researched in biomedical applications, including cancer treatment [121]. Graphene-based nanomaterials can be classified into several types depending on their composition, structure, and properties: single-layer graphene, multi-layer graphene, graphene oxide (GO) and reduced graphene oxide (rGO) [122]. Graphene has unique electrochemical and mechanical properties, for example, optical transmittance, chemical inertness, high density, molecular barrier abilities and high hydrophobicity [123, 124]. Graphene also has other remarkable features that contribute to cancer theragnostic such as high planar surface enabling higher drug-loading capacity [125] and thermal conductivity (5000 W/mK) [126]. However, van der Waals forces and *π*–*π* stacking interactions cause poor solubility and agglomeration of nanosheets formed by graphene in solution, which significantly affects toxicity and hampers its fabrication [127, 128]. These drawbacks have driven researchers to look for more bioavailable graphene-based nanomaterials that retain graphene’s advantages while being easy to fabricate. GO is a chemically modified material based on graphene. Functional oxygen groups such as carboxyl (–COOH) and Hydroxyl (C–OH) locate at the edge of graphene, while carbonyl (C=O) and epoxy groups (C–O–C) locate on the basal plane, thereby forming a typical GO molecule [125]. A rGO is the reduced derivative of GO. Compared to graphene, GO and rGO have improved properties regarding to biological usage. Defective oxygen-bound sp3 carbon atoms exhibit strong hydrophilicity and help forming of dispersions in aqueous solvents that are highly stable colloidal, preventing uncontrolled van der Waals, hydrophobic interaction induced aggregation [129]. Meanwhile, hydrophilic functional groups on the GO surface make the nanosheets a versatile platform for conjugating of various materials, which provides great potential in targeted therapy, PDT, PTT, and cancer diagnosis [130, 131].

Compared to other nanomaterials, graphene shows direct immunogenicity toward the immune system, and lateral size can regulate the extent of immunostimulatory capability both in vitro and in vivo [132]. Research shows that graphene activates the main components of the human immune system, macrophages and dendritic cells, indicating its potential in cancer treatment. Feito et al. studied the effect of the GO nanosheets specifically designed for hyperthermia cancer therapy on macrophage and lymphocyte function. The result showed that the 6-armed GO (6-GOs) significantly increased secretion of tumor necrosis factor alpha (TNF-α) by RAW-264.7 macrophages without changing IL-6 and IL-1β levels. In the presence of concanavalin A, lipopolysaccharide and anti-CD3 antibody, treatment of primary splenocytes involved 1-GOs and 6-GOs leading to significant dose-dependent cell proliferation and a decreased IL-6 level, which suggested the inherent weak inflammatory properties of GOs that are favorable for hyperthermia cancer therapy [133]. Graphene has also been found to inhibit some tumor cells. Burnett [134] treated human osteosarcoma (OS) cell and normal osteoblast cell with GO, and found that the apoptosis rate of OS cells was significantly higher than that of hFOB1.19 normal osteoblast cells. GO showed significant effects on cytotoxicity against OS, Nrf-2 decrease, ROS and cytomorphological changes. CSCs are generally considered a cancer cell population of high tumorigenic potency with self-renewable ability. CSCs interact with the TME and are believed to be involved in MDR formation [135]. Destruction of CSCs is one of the therapeutic approaches to avoid malignancy. It has been claimed that GO can specifically target CSCs rather than normal cells, and by inhibiting several key signaling pathways including WNT, Notch and STAT-signaling, GO induces CSC differentiation and inhibits tumor-sphere formation in multiple cell lines including breast, ovarian, prostate, lung, pancreatic and glioblastoma [136]. The researchers named this phenomenon, “differentiation-based nano-therapy”. However, few studies have been conducted over the past years, and more evidence may be needed. The interaction of graphene-immune cell interaction, effect graphene casts upon immune system and the direct anti-CSC phenomenon require further research.

As a nanomaterial with a high surface-to-volume ratio and plenty of oxygen-containing branches, graphene is a suitable platform for drug delivery, PDT, PTT. A GO-peptide hybrid was fabricated via irreversible physical adsorption of the Ac-(GHHPH)4-NH2 peptide sequence known to mimic the anti-angiogenic domain of histidine-proline-rich glycoprotein (HPRG). The hybrid nanomaterial was tested in prostate cancer cells (PC-3), human neuroblastoma (SH-SY5Y), and human retinal endothelial cells (primary HREC). The results showed that this GO-peptide nanoassembly effectively induced toxicity in the prostate cancer cells, blocked the cell migration, and inhibited prostaglandin-mediated inflammation in PC-3 and HRECs. Since poor nucleation, internalization of liposomal doxorubicin (L-DOX) limited its application in breast cancer, a novel DOX-loaded GO nanocarrier was created. The GO-DOX exhibited much higher anticancer activities when administered to cellular models of breast cancer. Through live-cell confocal imaging and fluorescence lifetime imaging microscopy, researchers found that GO-DOX achieved its high efficacy by inducing massive intracellular DOX release when bonded to the cell plasma membrane [137]. Many research indicates that GOs and rGOs can target at hypoxia [138] and abnormal angiogenesis in cancer TME [139, 140]. GOs and rGOs are also widely used in PDT and PTT [141, 142]. GDY is an allotrope of graphene that contains two acetylenic linkages in each unit cell, which double the length of the carbon chains connecting the hexagonal rings [143]. As a result, GYD is softer than either graphyne or graphene. In the past three years, several studies have been conducted using GYD as a drug delivery platform for photothermal/chemotherapy combinatorial approach in cancer diagnosis [144–146].

Fullerenes are molecules composed of carbon allotropes. The conformation of fullerenes includes hollow sphere, ellipsoid, or tube. Typical fullerenes include C60, C70, C82, etc. Metal atoms can be incorporated inside and form a metallofullerene [117]. Metal atoms encapsulated in the fullerene are usually Group III transition elements or a lanthanide. Since electrons of the intra-fullerene can transfer from encapsulated metal atom to the fullerene cage, metallofullerenes can be used as magnetic resonance imaging material. Properties of fullerenes also include free radical scavenging ability; therefore fullerenes can act as antioxidants [147, 148]. Compared to other nanomaterials, fullerene shows extraordinary properties in PDT and PTT. Chen et al. demonstrated that two critical factors leading to errors in photothermal efficiency estimation were laser irradiation time and nanoparticle concentration, and determined that photothermal conversion efficiency of polyhydroxy fullerenes was 69% [149]. The facts that the photothermal response of fullerenes remained stable with repeated laser irradiation, and the fullerene structure did not change, indicated that fullerenes were ideal candidates for photothermal therapy. A near-infrared (NIR) light-harvesting fullerene-based nanoparticles (DAF NPs) was tested for photoacoustic (PA) imaging-guided synergetic tumor photothermal and PDT. Compared to fullerene and antenna nanoparticles (DA NPs), DAF NPs showed better reactive oxygen species and heat generation efficacy. In vitro and in vivo studies demonstrated that DAF NPs could effectively inhibit tumor growth through synergetic PDT and PTT [150]. As a nanocarrier, fullerene has also been used in chemical drug delivery combined with PDT or PTT [150, 151].

CNTs are cylindrical tubes formed by sp2 -hybridized carbon atoms considered as rolls of graphene. The size of CNTs can vary from 1 nm to several micrometers. According to the number of layers formed in a CNT, CNTs can be divided into single-walled carbon nanotubes (SWCNTs) and multiwalled carbon nanotubes (MWCNTs). Poor water solubility and toxicity are two drawbacks of CNTs. Many studies on surface functionalization and material modifications have been carried out to solve the above problems and make CNTs more bioavailable. As a carbon-based nanomaterial, CNTs can interact with immune cells and induce immune response, therefore elevate immunity to suppress tumor growth [152, 153]. As a nanocarrier with a long research history being researched, CNTs are commonly considered an efficient PDT and PTT vehicle. Sundaram and his co-workers [154] coupled SWCNTs with hyaluronic acid (HA) and chlorin e6 (Ce6), and test this novel material in colon cancer cells using PDT. After 24 h, cellular changes were observed via microscopy, LDH cytotoxicity assay, and cell death induction. The result showed that the synthesized material enhanced the ability of PDT. Another synthesized NIR active photothermal agent, CNTs-PAMAM-Ag2S, was found to be highly efficient in PTT. The experiment showed that under irritation with 980 nm laser, photothermal efficacy of this complex was higher than that of copper-based and popular gold photothermal agents. Moreover, the complex demonstrated excellent stability against photo-bleaching and photo-corrosiveness, indicating the novel nanoagent could be promising in PTT [155].

Drug delivery systems (DDSs) based on CNTs loaded with DOX, PTX [156], cis‑platinum (CDDP) have been intensively studied [157–159]. CNHs belong to the carbon allotrope family. The conical structure is usually between 2 and 5 nm in diameter and the length of the larger spherical superstructures forming with sp2 hybridized carbon atoms is typically around 100 nm, which partly resembles the CNTs [160]. Similar to CNTs, CNHs lack solubility and require surface modification to be a nanocarrier in human tissue. Solutions include adding organic species onto the outer skeleton [161], conjugate planar aromatic molecules through electrostatic association or *π*–*π* stacking interactions [162, 163]. CNHs possess both drug-loading and photothermal abilities and were used in design of DDS with combined characteristics. Yang et al. made a dual chemo drug-loaded single-walled CNHs system. SWNHs were modified with poly and mPEG-PLA via hydrophobic-hydrophobic and *π*–*π* stacking interactions. Cisplatin and DOX were loaded onto modified nanohorns separately. The nanocarrier exhibited loading ability and efficient photothermal ability with a pH-dependent releasing capacity. Results showed that both primary breast tumors and the lung metastases were eradicated [164]. CNHs can also be modified with specific targeting molecules and applied in target chemical therapy. A cisplatin loaded CNH attached with a mAb D2B, selective for prostate specific membrane antigen (PSMA) + prostate cancer cells, showed superior efficacy and specificity to kill PSMA + prostate cancer cells compared to hybrids Ab-CNHs and cisplatin-CNHs [165].

Toxicity and side effects of CNMs used in cancer therapy have been studied in depth. Serum protein adsorption, hemolysis, cytotoxicity, and immunotoxicity have been reported for GO and rGO (93). As GO and rGO have a large surface area, they can be substrates for protein adsorption in the biological environment [176, 177]. With proteins adsorbed to the nanomaterial, loss of designed function and blockage of blood capillary might occur. In vitro and animal experiments indicated that the dose and size of GO and rGO could affect the toxicity of nanomaterials [178]. One study showed that large amounts hydrophobic rGO, accumulated on cell membrane, could induce high ROS stress and eventually lead to cell apoptosis [179]. In vivo studies revealed that CNTs could elicit chronic inflammation, granuloma formation, fibrosis, along with mesothelioma-like pathology [180]. Yan et al. summarized factors influencing CNT-induced toxicity such as surface modification, degree of aggregation, concentration, CNT size and shape, and listed up sites of CNTs accumulation after separation from anticancer drugs, which eventually suffer from CNT toxicity [181]. However, among this vast evidence achieved from various cells and animals, which aspect of CNMs plays a central role and the exact mechanisms of cellular toxicity caused by CNMs remain to be addressed [182].

### Quantum dots

Quantum dots are widely researched biomedical imaging probes due to their distinctive optical and electronic characteristics. They are typically nanometer-scale semiconductor crystallites and are broadly used to improve the efficacy of fluorescent markers in biological imaging [183]. Compared with organic fluorophores, QDs possess unique optical and electronic properties such as size and composition leading tunable fluorescence emission from visible to infrared wavelengths, large absorption coefficients, and high brightness levels photostability [184]. There are three common QDs based on carbon: graphene quantum dots (GQDs), nanodiamond and CDs. The most common use of carbon QDs is bioimaging, which can be applied to cancer imaging and sensing. GQDs are considered emerging nanomaterials in biosensing and cancer therapy because of the inherent grand surface suitable for molecular conjugation, superior biocompatibility, and rapid excretion. A photoluminescent glycodendrimer with terminal β-cyclodextrin molecules system was designed and used for DOX delivery with biocompatibility and pH-sensitivity. GQDs were used to provide the surface for PAMAM to grow from. After excitation at 365 nm by UV light, the emission spectra from GQDs and GQDs-PAMAM-β-CD were recorded. The result showed higher efficiency in killing cancer cells than that achieved by DOX alone and containing the GQDs made it a potential imaging agent with photoluminescent activity [185].

The fluorescent ability of GQDs was also used in a novel nanocarrier for targeted therapy. Researchers conjugated folic acid to sulfur-doped graphene quantum dots (FA-SGQDs) through simple pyrolysis of citric acid (CA), FA and 3-mercaptopropionic acid (MPA). The complex exhibited blue fluorescence with an emission band at 455 nm upon excitation at 370-nm wavelength, and a non-immunogenic FR-mediated endocytosis process for TA-SGQDs to enter the FR-positive cancer cells was revealed. In addition to bioimaging and biosensing, GQDs were also being investigated for PTT and PDT. A specifically modified GQD which exhibited strong absorption (1070 nm) in NIR-II region was prepared. The so-called 9T-GQDs having uniform size distribution, tunable fluorescence, and high photothermal conversion efficacy (33.45%) made it effective for ablating tumor cells and thus inhibited tumor growth under NIR-II irradiation, showing the potentiality of GQDs in PTT [186]. A combined photodynamic-chemotherapy DDS was designed based on carbon quantum dots. Researchers conjugated 5-aminolevulinic acid (5-ALA) with mono-(5-BOC-protected-glutamine-6-deoxy) β-cyclodextrin (CQD-glu-β-CD) moiety, and these materials were conjugated to CQDs loaded with DOX. High cytotoxicity and morphological changes of MCF-7 cancer cells were observed; also, ROS were induced by 15 min 635 nm (25 mW cm^−2^) radiation and achieved higher therapeutic effects [187]. CDs and nanodiamond have also been studied in cancer treatment utilizing its function of targeted therapy [188–190], PDT [191], cancer imaging [192] and antitumor immunity mediation [193, 194]. Compared with other carbon materials, research on carbon QDs is in its rising stage. Major obstacles in clinical translation of QDs are lack of standard protocol in high-quality QD production and their exact reaction mechanism and formation process [195].

### Metallic and magnetic nanomaterials

**Fig. 2:**
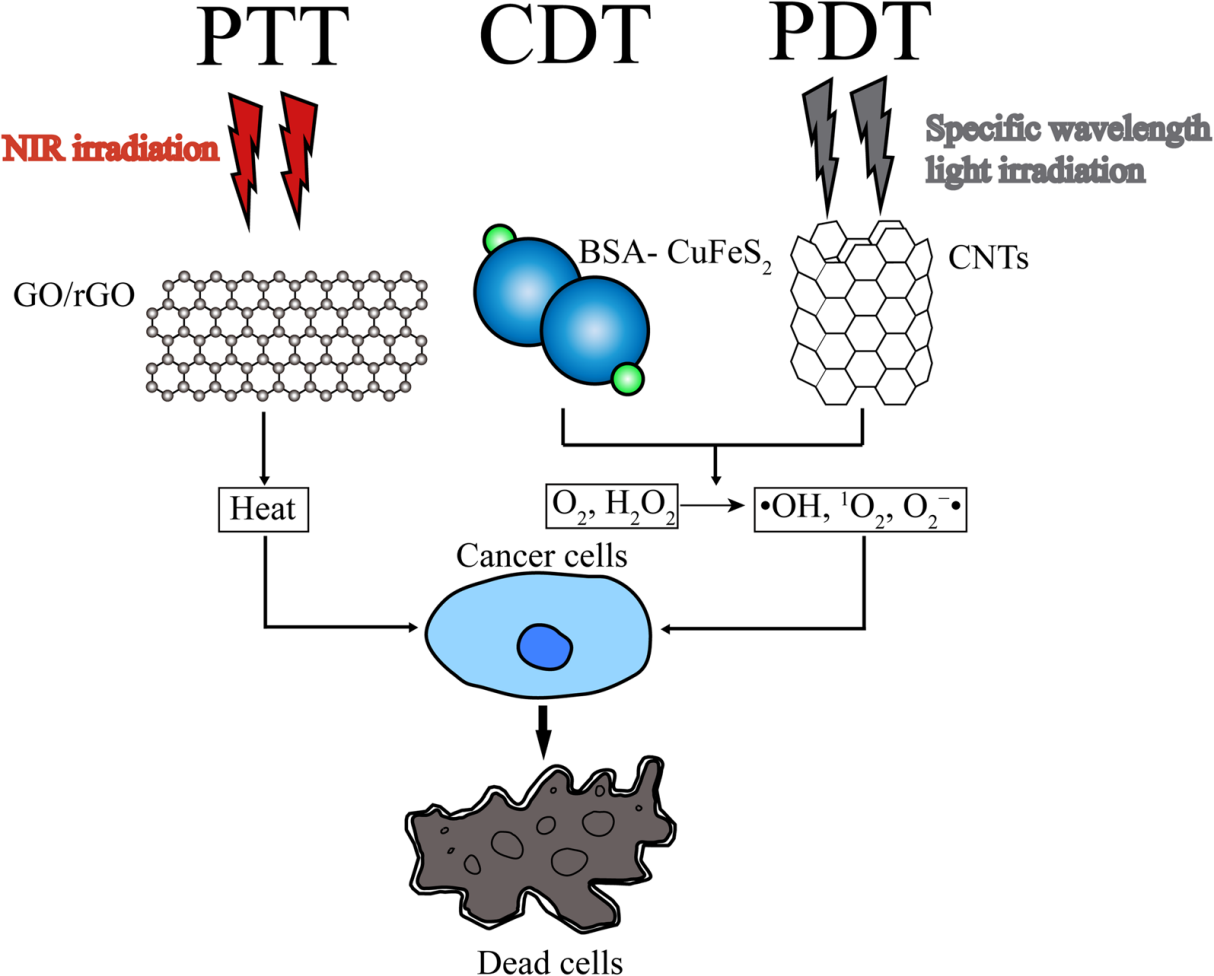
Schematic illustration of nanomaterial involved PTT, CDT, PDT. With NIR irradiation, PTT materials such as GO/rGO generate heat and cause cancer cell death. CDT material BSA-CuFeS_2_ and specific wavelength light irradiated PDT material CNTs generate ·OH, ^1^O_2_, O_2_ − · from O_2_, H_2_O_2_ in cells and cause cancer cell death. CDT, chemodynamic therapy; CNT: carbon nanotube; GO: graphene oxide; NIR: near-infrared; PDT: photodynamic therapy; PTT, photothermal therapy; rGO: reduced graphene oxide

**Fig. 3:**
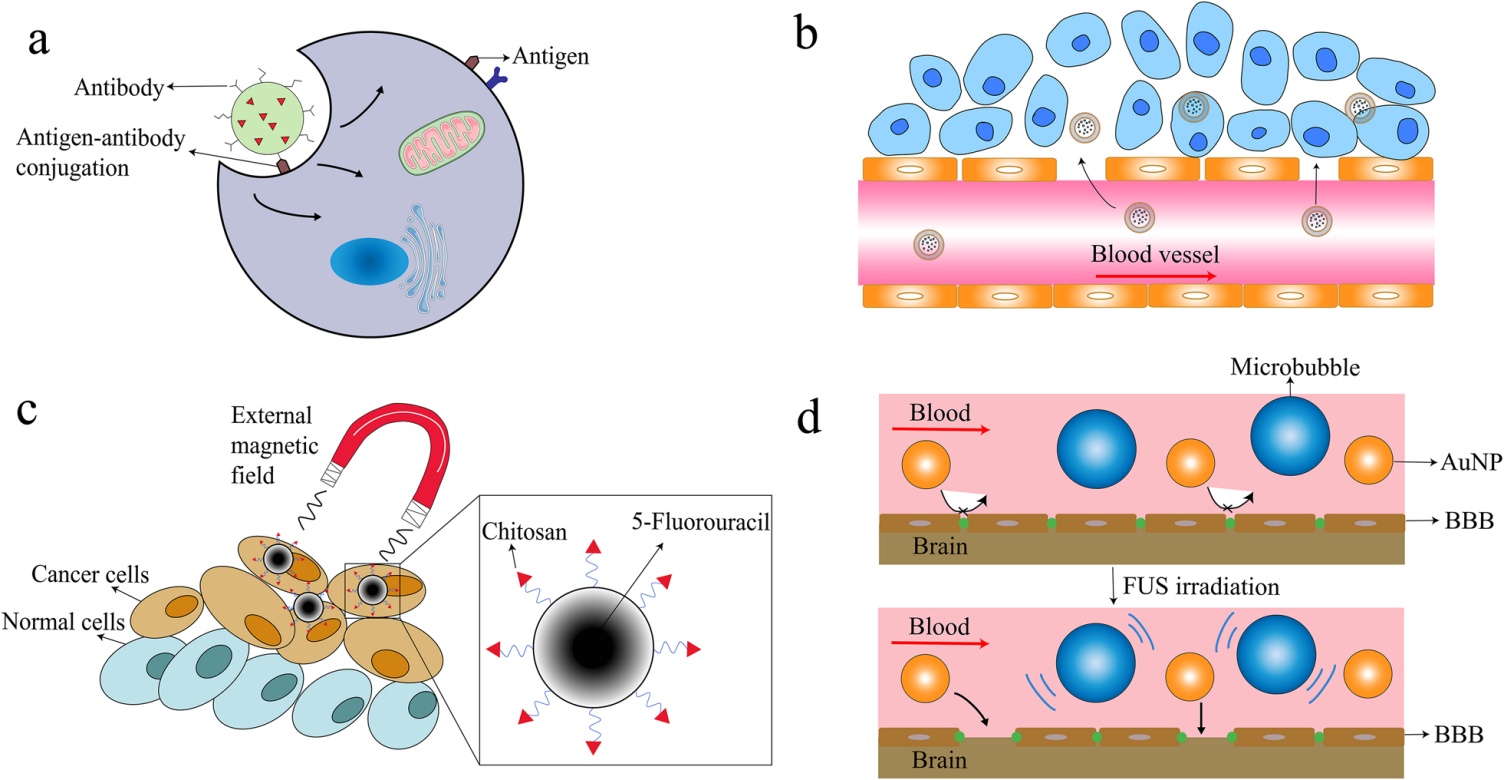
Illustration of interaction between nanomaterials and tumor cells. a Antigen–antibody conjugation modified nanoparticle endocytosis and transcytosis; b Liposome reaches cancerous area from blood vessels through EPR effect. c The magnetic nanoparticle coated with chitosan carries 5-Fluorouracil. Under external magnetic field, the nanoparticle shows passive targeting ability at cancer cells. d Therapeutic AuNP is blocked by BBB under normal status. After FUS exposure, the BBB is opened temporarily by microbubble inertial or stable cavitation and allows AuNPs to get through. BBB: blood–brain barrier; EPR: enhanced permeability and retention; FUS: focused ultrasound

Metallic nanoparticles have been extensively studied in bio-imaging and drug delivery because of their distinct optical, magnetic, and photothermal features. Metallic materials can be used in many forms in conjugation with versatile carriers such as NPs, liposomes, dendrimers or CNMs. Magnetic nanomaterials are mainly applied in MRI imaging. Guided by external magnetic field, magnetic NPs loaded with chemical drugs can target cancer cells, and therefore side effects of conventional chemotherapy are reduced [196] (Fig. 3). With metal particle conjugated, the nanosystem possesses both bio-imaging and PTT function. Iron oxide nanoparticles (IONPs) consisting of Fe_3_O_4_/Ag were encapsulated with a gold shell. MRI contrast capability was showed from IONPs and PTT due to the gold shell in the NIR region [37]. In cancer treatment, metallic materials are widely used in PTT, PDT, CDT, and immunotherapy. CDT is a Fenton or Fenton-like reaction-based therapeutic modality that relies on nanocatalyst [197]. Similar to PDT, highly oxidative hydroxyl radicals (·OH) are produced and toxic ·OH radicals take effect in cancer cells by triggering chain reactions with surrounding organic molecules, eventually leading to irreversible damage to DNA, lipids, and proteins [198]. During the process, iron-based nanostructures including FeS_2_, Fe_2_P, Fe_3_O_4_, SnFe_2_O_4,_ and amorphous iron are used to catalyze disproportionation of H_2_O_2_ to generate ·OH radicals [38, 199, 200]. For PTT and PDT, as NIR possesses much stronger tissue penetration ability than ultraviolet (UV) and visible light, NIR triggered materials are crucial in these therapies. In PTT, cancer cells are eliminated to the generation of thermal energy, while ROS including ·OH, singlet oxygen (1O_2_), and superoxide (O_2_ − ·) induce cytotoxic reactions in PDT [201]. Au (gold), Cu (copper), Fe (iron) are commonly used metallic materials in these therapies [202–204] (Fig. 2). The disadvantage of metallic nanomaterials lies in their toxicity. Attarilar et al. summarized the mechanisms of metallic NPs: ROS generation and influence on cell structures, characteristics of metallic NP toxicity are similar to other NPs, that toxicity is related to size, shape, dimensionality, surface charge [205]. Therefore, metallic NPs should be carefully examined before use on human patients.

## Cancer treatment and nanomaterial design

### Approaches in cancer treatment

To date, several mainstream approaches toward cancer treatment have been broadly applied to tackle cancer. Moreover, despite differences in working platforms, functional ingredients, and mechanisms, most researchers adopt two main targets: tumor cells and TME which include the immune system related to the tumor (Fig. 3).

#### Strategies targeting cancer cells

Targeting cancer cells is a natural method to eliminate cancer. With EPR and active targeting, modified nanocarriers such as NPs, dendrimers, or CNMs can reach cancer cells and release chemical drugs or biomaterials [206, 207]. Antibodies targeting specific antigens overexpressed on cancer cell surfaces are widely used in these platforms. After endocytosis by cancer cells, encapsulated chemical drugs exert cytotoxicity or nucleic acid materials induce cell apoptosis, depending on the encapsulated cargo. Progress has been made in nucleic acid delivery and nano-DDS based on exosomes [72, 78], PNPs, liposomes [208], dendrimers [115] are massively researched in cancer therapy.

#### Strategies targeting TME

**Fig. 4:**
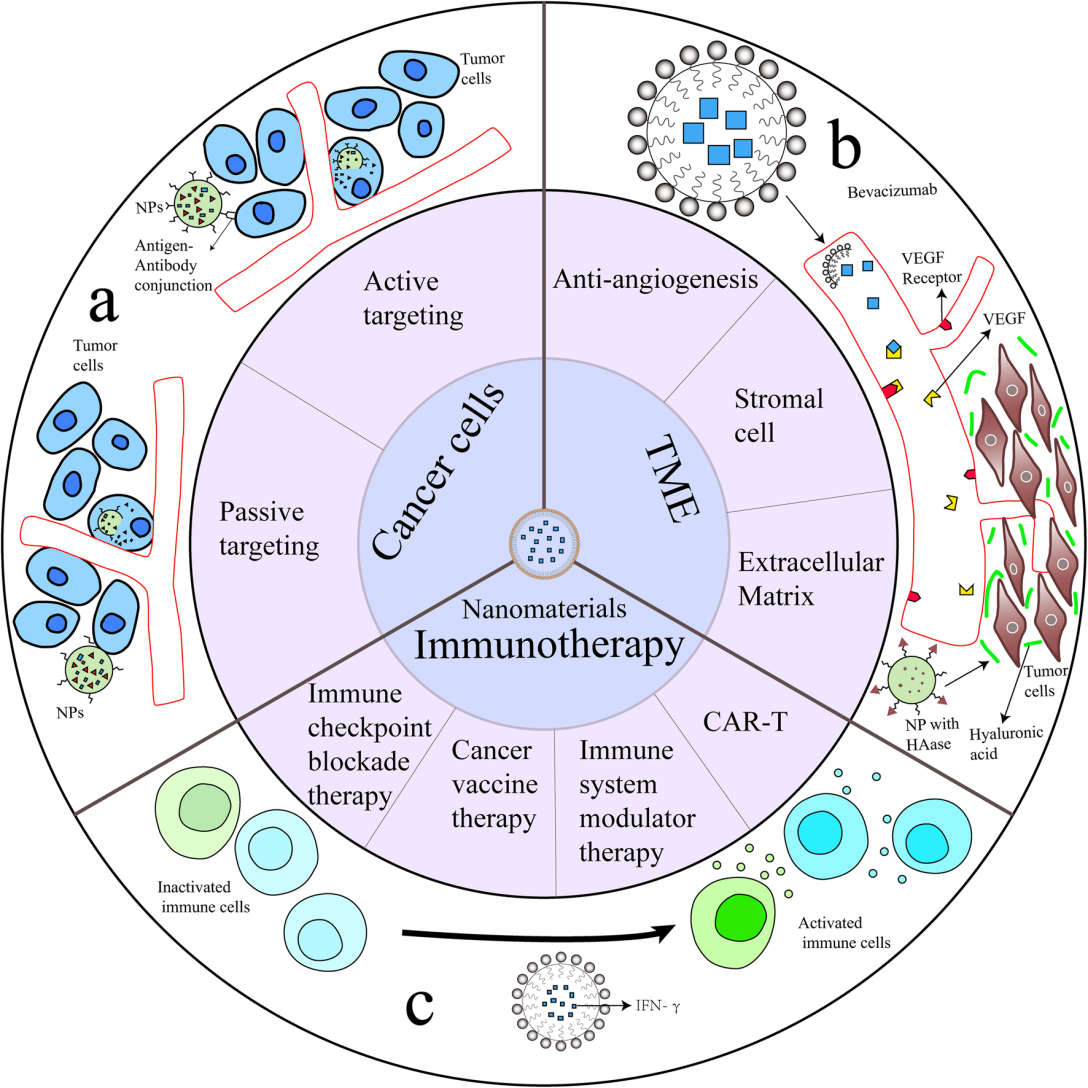
Cancer treatment approaches based on nanomaterials. **a** Targeting cancer cells by passive targeting or active targeting. **b** Targeting TME including anti-angiogenesis, stromal cell and extracellular matrix. Bevacizumab was loaded in liposome and conjugated with VEGF to inhibit angiogenesis. HAase was modified onto NP surface and enhanced NP penetration ability. **c** IFN-γ as an immune modulator delivered by liposomes activated immune cells in cancer immunotherapy. HAase: hyaluronidase; IFN-γ: Cytokine Interferon gamma; NP: nanoparticle; TME: tumor microenvironment; VEGF: vascular endothelial growth factor

Another strategy is about the TME that contain tumor cells. As mentioned above, angiogenesis is extremely active in almost all tumors because of uncontrolled cell proliferation and massive energy is needed for that. Research on this specific characteristic showed promising results. Sengupta designed a NP system specifically targeting abnormal tumor angiogenesis with combretastatin, and this medicine was co-encapsulated into the PLGA core with DOX. As a result, the DOX was efficiently taken up by the tumor after a rapid shutdown of the cancerous vessels induced by combretastatin, and an improved overall therapeutic index was achieved along with reduced toxicity [209]. In addition to abnormal vasculature, extracellular matrix (ECM) has also been researched in cancer treatment. ECM acts as a guiding scaffold in cancer proliferation, migration, invasion and angiogenesis [210]. Several main materials contributing to these cancerous properties are collagen, HA, various enzymes. As the main structural protein of the ECM, collagen forms migration tracks for tumor cells, while HA contributes to high interstitial fluid pressure (IFP), preventing drug diffusion and penetration [211, 212]. Enzymes, for example, matrix metalloproteinases (MMPs), can regulate TME by manipulating the activity of non-ECM molecules, including growth factors, receptors, and cytokines [213]. In nanocarrier design, ECM is one of the factors to be considered. Combined with conventional chemical drugs, recombinant human hyaluronidase (PEGPH20) in PEGylated form that targets at ECM hyaluronic acid exerted therapeutic effects for metastatic pancreatic cancer patients, especially in those with high hyaluronidase expression [214]. Efforts have been made to enhance chemical drugs loaded nanocarrier penetration ability in solid tumors by coating carriers with hyaluronidase (HAase) (Fig. 4b). This simple but effective strategy shows better anti-tumor efficacy [215].

#### Nanomaterials and cancer immunotherapy

The immune system plays a vital role in cancer formation and progression. There are several approaches in immunotherapy including immune checkpoint blockade therapy, chimeric antigen receptor (CAR)-T cell therapy, cancer vaccine therapy and immune system modulator therapy [216]. In these cancer immunotherapies, natural molecules or synthetic molecules are used to enhance or restore immune system function and exert anti-tumor effect. Programmed cell death protein 1 (PD-1) and programmed cell death ligand 1 (PD-L1) are important immune checkpoints and immune check point inhibitors (ICIs) targeting PD-1/PD-L1 has been researched to be loaded in nanocarriers targeting cancer [217]. In a research conducted by Bu and colleges, over-expression of PD-1 was considered to allow cancer cells to perform antitumor immunity evasion, and traditional immune checkpoint inhibitors (ICIs) of PD-1/PD-L1 showed inconsistent benefits. To ensure bonding of PD-L1 and ICIs, multivalent poly (amidoamine) dendrimers were employed; as a result, PD-L1 blockade effect was improved, and tumor site drug accumulation was enhanced [218]. CTLA-4 (cytotoxic T-lymphocyte-associated protein 4) is as an immune checkpoint with the function to downregulate immune responses [219]. Among these molecules are antibodies, small molecular inhibitors, proteins. Nanomaterials play an important role as drug vehicles to deliver these moieties (Table 4). Through these strategies, novel nanoplatforms can be developed and might achieve better efficacy and bioavailability than conventional therapies (Fig. 4) [106, 134].

**Table 4:** Nanomaterials applied in cancer immunotherapy

Name of drug	Component	Cancer types tested	Outcome	References
G7-aPD-L1	Dendrimer and anti-PD-L1 antibody	Human renal carcinoma and breast cancer cells	G7-aPD-L1 showed significantly enhanced binding strength to PD-L1 proteins compared to free aPD-L1	[218]
Doxil synergized with mAbs	Liposomal doxorubicin, anti-PD-1 and CTLA-4 mAbs	Mouse colon cancer cells and mouse fibrosarcoma cells	Doxil synergized with anti-PD-1 and CTLA-4 mAbs in a preventative CT26 mouse tumor model and Doxil activity increased in the presence of a functional immune system	[220]
NP_siCTLA-4_	Nanoparticle, CTLA-4 siRNA	B16 melanoma mouse model	NP_siCTLA-4_ delivered CTLA-4-siRNA into tumor sites and affected T cell subsets, exhibiting augmented T cell activation. Induced anti-tumor immune responses	[221]
NP-based mRNA vaccine	Nanoparticle, mRNA encoding tumor antigen Mucin-1	Mouse triple negative breast cancer cells and female BALB/c mice	The NP-based mRNA vaccine successfully expressed tumor antigen in mouse lymph node and a synergic anti-tumor effect was shown	[222]
rGO/MTX/SB	rGO, MTX, transforming growth factor beta inhibitor SB-431542 (SB)	Triple negative breast cancer mouse model	A synergistic chemo-immuno-photothermal anti-tumor effect by in situ vaccination and TME inhibition was exhibited after laser irradiation	[223]
IFN-γNE2	Nanoemulsion, IFN-γ	Human breast cancer cells	IFN-γNE2 reduced MCF-7 cell viability without affecting phagocytes and induced cellular activity of phagocytes	[107]

CTLA-4, Cytotoxic T-lymphocyte-associated protein 4; mAbs, Monoclonal antibodies; MTX, Methotrexate; IFN-γ, Cytokine Interferon gamma; NE, Nanoemulsions; PD-L1, Programmed cell death ligand 1; rGO, Reduced graphene oxide

### Advantages and challenges of nanomaterial applications in cancer therapy

Nanomaterials applied in cancer therapy have advantages over conventional chemical drugs as well as challenges in application. Several significant hallmarks in tumorigenesis and tumor development have been elucidated: continuous proliferative signaling, growth suppressors evasion, cell death resistance, replicative immortality, induced angiogenesis, activating invasion and metastasis, inflammation, genomic instability, and mutation [224, 225]. Traditional chemotherapy and radiotherapy have disadvantages in efficacy and side effects because of unspecific distribution and indiscriminate cytotoxicity to cancer cells and normal cells. Therefore, a delicate balance of dosing and an advanced targeting DDS is of great importance in cancer treatment [226]. To reach cancerous target sites, chemical drugs taken orally or intravenously shall pass several “fortifications”: TME and vasculature, MPS, BBB and kidney filtration. In physiological conditions, barriers like normal tissue microenvironment, vasculature, RES, BBB, and kidney filtration contribute significantly to pathogen resistance. However, in cancer treatment, intake of anticancer chemical drugs is affected by these defenses. Cancer cells hold a different proliferation pattern than normal cells. Cancer tissues exhibit distinctly in the dense extracellular matrix, over-activated angiogenesis induced by excessive angiogenic factors and high interstitial fluid.

#### Nanomaterial and drug metabolism

Drug metabolism is a complex process. MPS, also called as reticuloendothelial system or macrophage system [227], consists of blood monocytes, tissue macrophages, and other immune cells. When dealing with extrinsic molecules, in this case, chemical drugs, parts of the MPS such as immune cells in the liver, spleen, or lungs will react, and activated macrophages or leukocytes quickly eliminate the drugs, causing short drug half-life [228]. Nanocarriers with surface modification such as PEG or specific peptide possess lower MPS clearance and therefore prolong drug half-life [229]. Kidney filtration is an essential function of the renal system. Renal clearance rate associates with several properties, including particle size, shape, and surface charge. For traditional chemical drugs, renal clearance is one of the key points needed in drug delivery [230]. Proper renal clearance helps to minimize toxicity of nanocarrier. These barriers are obstacles for many conventional drug deliveries, diminishing drug efficacy in cancerous sites and indirectly increasing dosage and toxicity for normal tissue.

#### Nanomaterials and BBB

The BBB is a highly specialized protection structure that protects the central nervous system from harmful agents and provides essential nutrition. BBB consists of brain capillary endothelial cells, which are arranged to form a “wall.” Due to the blocking function of BBB, current post-surgery chemotherapy methods for brain cancer are mainly intraventricular or intracerebral direct injections, infusion, even implantation. However, these methods aiming at increased permeability might result in risks associated with high toxicity or inadequate drug distribution, that demands for a better solution to deliver anticancer drugs through BBB [231]. In brain tumor treatment, conventional free chemical drugs are hard to reach cancerous sites through intravenous method due to BBB, and nanomaterials are researched to overcome this obstacle. EPR effect, peptide-modified endocytosis and transcytosis, focused ultrasound (FUS) are major approaches currently utilized to help deliver nanomaterials (Fig. 3). Several nanomaterials have been researched for delivery through BBB, including NLCs [232], liposomes, and AuNPs. A glutathione PEGylated liposome loaded with methotrexate (MTX) was tested in rats and the result showed the nanocarrier improves the brain uptake of MTX [233]. AuNPs are vastly researched among these materials. Research concerning glioma and other intracranial cancers are conducted mainly in the mouse model, and the result shows that EPR effect allows gold nanoparticles (AuNPs) of certain size to accumulate in the tumor [234]. To gain better specificity, inorganic NPs such as AuNPs can also be modified with peptides and antibodies on the surface. AuNPs and gold liposomes are used as biocargo for chemical drugs and nucleic acid, and AuNPs are also used in PTT and immune therapy. Ruan et al. fabricated a novel NP AuNPs-A&C-R that were composed of two functional particles, and both particles were peptide modified AuNPs. Peptide attached on the surface helps mediate AuNPs-A&C-R transcytosis across BBB and target receptors on glioblastoma cell surface. This AuNP loaded DOX and showed better chemotherapeutic effect than free DOX treatment [235]. Research indicates that ultrasound can widen BBB tight junction therefore offers a temporary pathway for NPs to get through, and size of AuNPs affects delivery efficiency. This research shows ultrasound might help AuNPs with therapeutic function to penetrate BBB with ultrasound treatment [236] (Fig. 3). Current mouse experiments show that modified AuNPs can help transport chemical drugs, induce lethal autophagy and apoptosis [237] and exert photothermal effect in intracranial cancer PTT [238].

#### Targeting strategies of nanomaterials applied to cancer therapy

Targeted therapy aims at specific biological pathways or proteins that function in tumor growth. Molecules related to apoptosis and angiogenesis are also common targets in targeted therapies. Small molecules inhibitors and mAbs are two major tools to be utilized in targeted therapies [14]. Through antigen–antibody conjugation, better specificity can be achieved. Compared with non-targeted therapies, free chemical drugs for example, targeted therapies specifically affect tumor-related molecular targets, while free chemical drugs kill both rapidly dividing normal cells and cancer cells. NPs loaded with targeted therapy drugs or modified with specifically targeted mAbs in the surface gain better efficacy and lower toxicity compared to nanocarriers loaded with anti-tumor chemical drugs (Table 1).

The EPR effect is a fundamental mechanism applied in nanocarrier targeting strategy. Passive targeting based on EPR effect involves interactions between the nanoplatform and TME, MPS, and barriers in the human body. It should be noted that EPR also functions in active targeting strategy achieved by conjugating with antibodies, peptides, aptamers and small molecules, and the efficacy of active targeting is affected by MPS, immune system, and other nanocarrier–environment interactions. Both passive targeting and active targeting strategies are used in DDS design. By loading the nanocarrier or modifying the surface with therapeutic ingredients in targeted therapy, the fabricated nanoplatform can be utilized to improve current targeted therapy and achieve better efficacy.

#### Current challenges of nano-DDS designing

Three key issues should be considered in anti-cancer nano-DDS designing: enhancement of efficacy, reduction of side effects, and resistance prevention. In many cases, a nano-DDS can solve several problems simultaneously due to instinct mechanism. A SLN synthesized with the material dexamethasone (Dexa)-conjugated lipid is linked with PEG-phosphatidylethanolamine (PEG-PE) and obtains Tf (transferrin)-PEG-PE ligands. As many cancer cells over-express the Tf receptor and use it to obtain certain molecular epitope, Tf is considered the target moiety that binds to the TfR molecular on the HepG2 cells [239]. This kind of surface modification makes it a better delivery vehicle for gene, and the experiment shows that it displays remarkably higher transfection efficiency than both non-modified SLNs/pEGFP and vectors that do not contain Dexa in vitro or in vivo [240]. The increased specificity results in higher drug accumulation in targeted cancer sites than other vital organs, leading to reduced toxicity and drug-related MDR prevention [241].

Despite rapidly growing research concerning nanomaterials in cancer treatment, some issues still remain unsolved. Toxicity is still one of the main concerns of nanomaterials. Because of the extremely small size, physiological barriers can be penetrated through, which may pose potential health hazards [242]. Evidence shows that cellular membranes, organelles, and DNA suffer from free radicals caused by NPs [243]. Nanomaterials delivered intracellularly might stimulate an immune response by reacting with cell surface receptors [244, 245]. As referred to above, nanomaterial toxicity relates to many factors and thus, modification to reduce toxicity is essential in the fabrication process.

As the primary passive delivery method utilizing nanomaterials, the EPR effect has been closely studied for a long time. However, most designed nanomaterials failed to reach the stage of clinical use. Some researchers tried to re-consider the concept of EPR and explore the real efficacy of this “royal gate” toward cancer treatment. The EPR effect works in rodents differently as in humans [246]. Sindhwani et al. investigated the mechanism by which NPs enter solid tumors. The experiments used four different mouse models, three types of human tumor cells, mathematical simulation and modeling, two imaging techniques, and the results were stunning. The frequency of gaps in tumors did not account for nanoparticle accumulation in tumor. Trans-endothelial pathways were the dominant mechanism of nanoparticle tumor extravasation. Finally, combined evidence from TEM and 3D microscopy showed that there were not enough gaps, which resulted in rare opportunities for cancer nanomedicine to enter tumors passively [247]. These studies indicate that the differences in EPR efficacy in various cells and tissues need further investigations. Studies have been conducted to stratify cancer patients by accumulating NPs through EPR and to find predictive EPR markers [248, 249]. These results indicate that the EPR effect varies in different species and tumors. To better exploit the EPR effect in cancer therapy, more research is needed to explore different patterns and efficiencies of the EPR effect and elucidate the mechanism of nano-carrier transport.

Another knotty obstacle of nanomaterial implementation in cancer treatment lies in clinical translation. Although plenty of nanocarrier research for cancer therapy has been conducted (Table 5), most of these researches involve cell and animal models that may not reflect coherent responses in actual human organs. A single model is hard to imitate real reaction in the human body, and previous studies exhibited more consistency of EPR in animals than in human patience [250]. Models of cancer metastasis should also be considered in research as metastasis is common for malignant cancers. The specific solution to these problems is hard to reach; however, innovative modeling methods can be explored to accelerate the process. Biomimetic ‘organ/tumor-on-a-chip’ tools, organoid model systems are possible solutions to imitate in vivo situation of nanocarriers used in cancer patients [251–253]. Proper animal models are also recommended in these assessments. Properties of nanomaterials, including size, shape, chemical composition, surface charge, have an enormous influence on nanocarriers' overall efficacy, and adjustment of these properties needs researchers' cooperation in both medicine and material fields. So far, approved nanocarriers used in cancer therapies are mostly liposomes and nanoparticles, and nanocarriers with more complex structures and manufacturing procedures generally face greater difficulties in clinical translation (Table 5). Searching for technology that helps manufacture vast nanomaterials with combined required properties is one important goal in anticancer nanomaterial clinical translation.

**Table 5:** Examples of nanocarriers for anticancer therapy

Nanotechnology platform	Description	Pharmaceutical ingredients	Disease	Status	References
PNP	Decorated with somatostatin analogue	Cetuximab	Colon cancer	Phase 1	NCT03774680
NP	Combined with enzalutamide	Camptothecin	Metastatic castration resistant prostate cancer	Phase 2	NCT03531827
NP	Co-coated drug	Nab-paclitaxel rituximab	B-Cell Non-Hodgkin Lymphoma	Phase 1	NCT03003546
Liposome	Liposome	irinotecan	Small cell lung cancer	Phase 3	NCT03088813
Liposome	Pegylated liposomal carrier	Doxorubicin trastuzumab	HER2-positive metastatic breast cancer	Phase 2	NCT03933319
Nanoemulsion	Photosensitizer in PDT therapy	Aminolevulinic acid nanoemulsion	Basal cell carcinomas	Phase 2	NCT02367547
Quantum dot	Coated with drug	veldoreotide	Breast cancer	Phase 1	NCT04138342
Albumin NP	NP bound albumin (Abraxane)	Paclitaxel	Breast cancer, NSCLC, pancreatic cancer	Approved by FDA	[254]
Liposome	Liposome (DepoCyt)	Cytarabine	Lymphomatous malignancies	Approved by FDA	[255]
Liposome	Liposome (Marqibo)	Vincristine sulfate	Acute lymphoblastic leukemia	Approved by FDA	[256]
Liposome	Liposome (Doxil)	Doxorubicin	HIV-related Kaposi sarcoma, ovarian cancer, multiple myeloma	Approved by FDA	[19]
Liposome	Liposome (DaunoXome)	Daunorubicin	HIV-related Kaposi sarcoma	Approved by FDA	[257]
Polymer protein conjugate	Multi-agent chemotherapy-eutic regimen (Oncaspar)	L-asparaginase	leukemia	Approved by FDA	[258]

NP, nanoparticle; PDT, Photodynamic therapy; PNP, Polymeric nanoparticle

US Clinical trials website (http://clinicaltrials.gov/) [259]–US Food and Drug Administration website (http://www.accessdata.fda.gov/) [260]

### Proteomics and anti-cancer nanoplatform design

When injected into a biological system, nanomaterials are surrounded by serum and cellular proteins, structures formed by these substances are termed protein corona (PC) [261]. Searching for technology that helps manufacture vast nanomaterials with combined required properties is one important goal in anticancer nanomaterial clinical translation. It has been found that since different binding affinities toward NPs are shown by proteins, “hard” corona can form with higher binding affinity proteins, while “soft” corona forms with proteins that bind loosely to nanoparticles. As a result, the most abundant proteins that form a PC first, with time they will be replaced by the proteins with higher affinities. This phenomenon is named as Vroman effect [262]. Various proteomic methods have been used in PC research, especially in quantitative analysis: MS, LC–MS, SDS-PAGE [263], surface plasmon resonance (SPR), isothermal microcalorimetry (ITC). PC affects the interaction of NP with biological environment and therefore, determines whether a NP carrier could be applied in medical use to a degree. Thus, proteomic methods help study NP-protein interaction and achieve a deeper understanding of PC formation.

Cancer proteomics analyzes protein quantity in cancer cells and serum, which helps find proteins and surface biomarkers useful in cancer diagnosis and prognosis [264]. Proteomics has also been applied to help understand cancer pathogenesis, elucidate the mechanism of drug resistance, and search for biomarkers for early detection of cancer [265]. In the pathological process, PTMs (post-translational modifications) are important mechanisms related to cancer occurrence, metastasis and reoccurrence, and kinase plays essential roles in these modifications and pathways. Although chemical drugs are the current focus of research, kinase inhibitors and other novel therapeutic agents such as siRNA, mRNA, and gene editing materials cognized through cancer proteomics approaches can be loaded within a nanocarrier to achieve higher drug efficacy. New molecular targets can also be identified by proteomic methods, enriching currently recognized targeting moieties. High throughput proteomics and many novel ways are also enhancing the capability of proteomic methods to identify specific molecules potential for manufacturing anticancer nanocarriers.

## Conclusions

Nanomaterials share similar size but differ in composition, structure, hydrophobicity, magnetism, immunogenicity and other properties. Cancer therapies based on these unique properties have been vastly researched. In general, various surface modification can be achieved on different nanomaterials, and in many cases, conventional anti-tumor chemical drugs can be loaded into different nanocarriers. It is crucial for researchers to be well aware of the characteristics of the selected nanoplatform as well as properties of therapeutic agents. For instance, EVs are biocompatible vesicles with ability to escape the immune surveillance and internalize smoothly with target cells, a possible strategy might be using antibody modified EV to deliver key gene therapy agents to targeted cancer cells. Based on photothermal properties CNTs and metallic materials possess, nanoplatform functions with chemotherapy and PTT can be designed to produce synergistic effect. CNTs have the potential to achieve better anti-tumor efficacy for the feature that they can provide several kinds of therapies at the same time. Both targeted delivery and non-targeted delivery employ nanomaterials as vehicles to transport chemical drugs, peptide/protein molecules, small molecule inhibitors or use the material as immune system stimulant, photothermal medium, chemodynamic medium. Modification of the nanomaterial platform including inner content and external moiety plays an important role in the efficacy, targeting ability, biocompatibility and toxicity of the nanoplatform complex.

In this article, we mainly focus on characteristics of common nanomaterials and progress of their application in cancer therapy rather than the chemical synthesis process and drug-loading technique which are also important issues limiting clinical translation of nanomaterials. Targeting therapy and immunotherapy that involve molecules in newly discovered pathways are being massively researched. It is expected in the future, with development in proteomic research on mechanism of cancer genesis, MDR occurrence, more nanomaterial-based targeting therapy and immunotherapy approaches will be explored.

Compared to the enormous amount of research, only a few nanomaterial-based drugs are applied in clinical. To improve this situation, more efforts should be taken into toxicity reduction, illumination of EPR and PC mechanism in the human body. It is expected that in the near future, nanoplatforms will be designed to target not only on cancer cells, but also on the TME environment including immune system. Precise targeting methods, TME triggered release strategy, combined therapies, self-assembly nanoplatform are practical approaches to enhance targeting specificity, drug capacity, efficacy, bioavailability; and reduce the toxicity of nanomaterials and loaded drugs toward normal cells. Testing nanomaterials in models that resemble more in vivo environment is also an important issue to be considered. Overall, with the advancement of nanobiotechnology and cancer therapy development, we believe that the breakthrough in clinical translation for treating cancer, a deadly disease, will be achieved, and more nanomaterial-based drugs will benefit cancer patients.

## Data Availability

Not applicable.

## Abbreviations

5-ALA: 5-Aminolevulinic acid
AA-PEG: Aminoethylanisamide-polyethylene glycol
ABCs: ATP-binding cassette transporters
ADCs: Antibody–drug conjugates
AuNPs: Gold nanoparticles
BBB: Blood–brain barrier
Bis-MPA: 2,2-Bis(hydroxymethyl) propionic acid
CA: Citric acid
CAR: Chimeric antigen receptor
Cas: CRISPR-associated proteins
CDP: Cyclodextrin polymer
CDs: Carbon dots
CDT: Chemodynamic therapy
Ce6: Chlorin e6
CL: Cationic liposome
CMCS: Carboxymethyl chitosan
CNHs: Carbon nanohorns
CNTs: Carbon nanotubes
CQDs: Carbon quantum dots
CRISPR: Clustered regularly interspaced short palindromic repeats
CSCs: Cancer stem cells
CTLA-4: Cytotoxic T-lymphocyte-associated protein 4
DDS: Drug delivery system
Dexa: Dexamethasone
DOX: Doxorubicin
ECM: Extracellular matrix
EPR: Enhanced permeability and retention
EVs: Extracellular vesicles
exoDOX: Exosomes loaded with DOX
FA-SGQDs: Folic acid to sulfur-doped graphene quantum dots
FNPs: Fluorescent polymeric nanoparticles
FUS: Focused ultrasound
GDY: Graphyne
GO: Graphene oxide
GQDs: Graphene quantum dots
GSH: Glutathione
HA: Hyaluronic acid
HAase: Hyaluronidase
HER2: Human epidermal growth factor receptor 2
HPRG: Histidine–proline-rich glycoprotein
HREC: Human retinal endothelial cells
ICIs: Immune check point inhibitors
IFN-γ: Cytokine Interferon gamma
IFP: High interstitial fluid pressure
IHC: Immunohistochemical
ING4: Inhibitor of growth 4
IONPs: Iron oxide nanoparticles
ITC: Isothermal microcalorimetry
L-DOX: Liposomal doxorubicin
LUV: Large unilamellar vesicles
mAbs: Monoclonal antibodies
MDR: Multi-drug resistance
MEK/ERK: Mitogen-activated protein kinase kinase/extracellular signal regulated protein kinase
miR-497: MiRNA-497
miRNAs: MicroRNAs
MLV: Multilamellar vesicles
MMPs: Matrix metalloproteinases
MPA: Mercaptopropionic acid
MPS: Mononuclear phagocyte system
MTX: Methotrexate
MWCNTs: Multiwalled carbon nanotubes
NE: Nanoemulsions
NIR: Near-infrared
NLCs: Nanostructured lipid carriers
NPs: Nanoparticles
NSTI: Nanoscience, technology, and industry
OS: Osteosarcoma
PA: Photoacoustic
PAMAM: Polyamidoamine
PC: Protein corona
PCL: Poly(ε-caprolactone)
PCX: Etoposide and paclitaxel
PD-1: Programmed cell death protein 1
PD-L1: Programmed cell death ligand 1
PDT: Photodynamic therapy
PEG: Poly(ethylene glycol)
PEG-PE: PEG-phosphatidylethanolamine
P-gp: P-glycoprotein
PI3K/mTOR: Phosphoinositide 3-kinase/mammalian target of rapamycin
PLA: Polylactic acid
PLGA: Poly(lactic-co-glycolic acid)
PMMA: Polymethyl methacrylate
PNPs: Polymeric nanoparticles
PPI: Polypropylenimine
PTEN: Phosphatase and tensin homolog
PTMs: Post-translational modifications
PTT: Photothermal therapy
PTX: Paclitaxel
rGO: Reduced graphene oxide
RGD: Arginine-glycine-aspartic
RNAi: RNA interference
ROS: Reactive oxygen species
SDT: Sonodynamic therapy
Sf: Sorafenib
Si: SiRNA
siRNA: Small interfering RNAs
SLNs: Solid lipid nanoparticles
SPION: Superparamagnetic iron oxide nanoparticles
SPR: Surface plasmon resonance
SUV: Small unilamellar vesicles
SWCNTs: Single-walled carbon nanotubes
TEA: Triethanolamine
Tf: Transferrin
TLR4/NF-κB: Toll-like receptor 4/nuclear factor kappa B
Tmab: Trastuzumab
TME: Tumor microenvironment
TNF-α: Tumor necrosis factor alpha
TPGS: Tocopheryl polyethylene glycol 1000 succinate
TRAIL: Tumor necrosis factor-related apoptosis-inducing ligand
UV: Ultraviolet
VEGF: Vascular endothelial growth factor
